# Handling of Doubtful WBC Scintigraphies in Patients with Suspected Prosthetic Joint Infections

**DOI:** 10.3390/jcm9124031

**Published:** 2020-12-13

**Authors:** Chiara Lauri, Giancarlo Lauretti, Filippo Galli, Giuseppe Campagna, Simone Tetti, Donatella Riolo, Alberto Signore

**Affiliations:** 1 Nuclear Medicine Unit, Department of Medical-Surgical Sciences and of Translational Medicine, “Sapienza” University of Rome, 00161 Rome, Italy; chialau84@hotmail.it (C.L.); giancarlolauretti@gmail.com (G.L.); filippo.galli@hotmail.com (F.G.); gius.campagna@gmail.com (G.C.); simrad@hotmail.it (S.T.); donatellariolo89@gmail.com (D.R.); 2 Department of Nuclear Medicine and Molecular Imaging, University of Groningen, University Medical Center Groningen, 9700 Groningen, The Netherlands

**Keywords:** WBC scintigraphy, qualitative analysis, semi-quantitative analysis, BMS

## Abstract

Despite the application of EANM recommendations for radiolabelled white-blood-cells (WBC) scintigraphy, some cases still remain doubtful based only on visual analysis. The aim of this study was to investigate the role of semi-quantitative analysis and bone marrow scan (BMS) in solving doubtful cases. We retrospectively evaluated all [^99m^Tc]HMPAO-WBC scintigraphies performed, in the last 7 years, for a suspected monolateral prosthetic joint infection (PJI). In doubtful cases, we used five different thresholds of increase of target-to-background (T/B) ratio, between delayed and late images, as criteria of positivity (5%, 10%, 15%, 20% and 30%). BMS were also analysed and sensitivity, specificity and accuracy of different methods were calculated according to final diagnosis. The sensitivity, specificity and accuracy were, respectively, 77.8%, 43.8% and 53.0% for the cut-off at 5%; 72.2%, 66.7% and 68.2% for the cut-off at 10%; 66.7%, 75.0% and 72.7% for the cut-off at 15%; 66.7%, 85.4% and 80.3% for the cut-off at 20%; 33.3%, 93.8% and 77.3% for the cut-off at 30%. BMS provided a significantly higher diagnostic performance than 5%, 10% and 15% thresholds. Conversely, we did not observe any statistically significant difference between BMS and the cut-off of more than 20%. Therefore, doubtful cases should be analysed semi-quantitatively. An increase in T/B ratio of more than 20% between delayed and late images, should be considered as a criterion of positivity, thus avoiding BMS.

## 1. Introduction

Joint prosthesis replacement is constantly growing due to the increase of life expectancy. Surgery aims at obtaining pain relief and improving joint functionality and quality of life (QoL), thus resulting in a significant social cost saving [1]. However, uncommon complications may occur such as mechanical loosening or prosthetic joint infection (PJI). The incidence of PJI ranges between 2.0% and 2.5% for primary surgical replacement [2] and up to 20% for revision procedures [3]. The improvement of surgical techniques, the use of more biocompatible prosthetic materials and the optimization of therapeutic management in the post-operative period have reduced the incidence of clinically evident infections that may be easily detected with conventional radiology and biochemistry. The infections are often indolent, sub-acute or chronic thus delaying and complicating the diagnosis and further management. As consequence, clinicians, Nuclear Medicine (NM) physicians and radiologists deal with more challenging and complex cases.

Recently, the European Association of Nuclear Medicine (EANM), European Bone and Joint Infection Society (EBJIS), European Society of Radiology (ESR) and European Society of Clinical Microbiology and Infectious Diseases (ESCMID) published a joint guideline on the diagnosis of PJI aiming at providing diagnostic flow-charts for its management [4]. Since each diagnostic approach has its pros and cons, as always, the correct diagnosis relies on the combination of medical examination, clinical history, laboratory tests, microbiology and histology and imaging modalities. The identification of the pathogen at microbiology still remains the corner stone for the differential diagnosis between aseptic loosening and PJI; however, bacterial cultures and leukocyte count from joint aspiration or, in alternative, biopsies are invasive procedures that may expose the patient to the development of an infection, and they may present false negative results during an antibiotic treatment [5,6,7,8]. Radiology is useful to detect potential abnormalities involving the implant and/or surrounding bone and soft tissues, but its modalities are usually not able to discriminate between an infection and an aseptic loosening, since typical radiological signs of infections may appear in late stages of the process [4,9]. Moreover, metal artefact may complicate the interpretation of Computed Tomography (CT) and Magnetic Resonance Imaging (MRI).

On the other hand, NM, with its functional imaging, offers several techniques and radiopharmaceuticals able to confirm or rule out the infection, even in the earlier stages, with high diagnostic accuracy [10,11]. In this view, radiolabelled white blood cells (WBC) scintigraphy, with both [^99m^Tc] and [^111^In], is able to accurately differentiate a PJI from a mechanical aseptic loosening [12]. Many efforts have been made by EANM in order to standardize labelling procedures [13,14], acquisition protocols and interpretation criteria [15], since the high accuracy of this modality relies on these crucial aspects. Once correctly acquired, with times corrected for isotope decay, the images have to be properly displayed by using a “total count” intensity scale and the same intensity threshold [15,16,17] in order to reduce the observer bias. The interpretation is usually based on visual analysis of the uptake over time in the affected joint: an increased uptake between delayed and late images, in terms of intensity and/or extension, is indicative of an infection. Conversely, a decreased/stable uptake over time rules out the infection with very high negative predictive value (more than 90%–100% depending on the studies) [15,16,17]. However, several factors, e.g., only a small change of uptake intensity or extent over time, or the presence of bone marrow (BM) interference that does not allow a correct evaluation of the affected bone, may complicate the visual analysis, thus making the diagnosis more challenging. When qualitative assessment is not sufficient, a semi-quantitative analysis has been proposed [18], comparing target to background (T/B) ratio between delayed and late images and using the contralateral tissue as reference [17], but a precise threshold of increase in T/B ratio over times has never been defined. It is well known that false positive results may occur in presence of physiologic BM expansion as a consequence of migration of WBC in a reticulo-endothelial system; therefore, a combination of bone marrow scintigraphy (BMS) and WBC scintigraphy is strongly suggested in order to improve the accuracy [19,20,21]. However, this approach requires additional radiations to the patients.

Therefore, the purpose of this study is to explore whether semi-quantitative analysis could be able to solve doubtful cases thereby reducing the number of BMSs and reserving this combined approach only to selected cases.

## 2. Experimental Section

### 2.1. Materials and Methods

We retrospectively evaluated all [^99m^Tc]HMPAO-WBC scintigraphies for suspected mono-lateral PJI performed in the NM Department in Sant’Andrea Hospital of Rome from January 2011 to December 2017. Amongst them, two independent NM readers (AS and CL), experts in infection and inflammation imaging, selected doubtful scintigraphies according to qualitative analysis. Discordant cases were reviewed by a third reader (GL) and resolved by consensus.

Doubtful cases were analysed with a semi-quantitative assessment by evaluating 5 different thresholds (5%, 10%, 15%, 20% and 30%) of increase in T/B ratio between delayed and late images, as criteria of positivity. BMS, when available, was also analysed in addition to semi-quantitative evaluation.

Sensitivity, specificity and accuracy of semi-quantitative analysis and BMS were calculated and were used to create a procedural algorithm useful in equivocal cases.

#### 2.1.1. Patients

Patients were retrospectively recruited in NM Department of our hospital. The following inclusion and exclusion criteria were adopted in this study:

Inclusion criteria (the three items were mandatory):

- Suspected mono-lateral PJI of hip or knee, according to IDSA criteria [22];
- Final diagnosis provided by gold standard;
- Equivocal WBC scintigraphy at visual analysis;
- BMS, if available;
- Raised inflammatory markers, if available.

Exclusion criteria:

- Clearly positive or negative scintigraphy at qualitative analysis;
- Lack of information on final diagnosis;
- Patients lost at clinical follow-up;
- Patients with suspected bilateral prosthesis;
- Ongoing antibiotic treatment at the time of WBC scintigraphy.

Demographic and laboratory data including gender, age, medical history, biochemistry, microbiology, histopathology, treatments and final diagnosis were collected.

#### 2.1.2. Acquisition Protocol and Image Display of WBC Scintigraphy

All [^99m^Tc]HMPAO-WBC scintigraphies were performed according to EANM guidelines for labelling, acquisition protocols and interpretation criteria [13,14,15], using Leukokit^®^.

Planar anterior-posterior (AP) views of the hips and AP plus medial-lateral views for knees, were acquired with times corrected for [^99m^Tc] decay, at three times point post injection (p.i.) of autologous leukocytes, with following parameters:

- early planar images (30 min: 1 h p.i.) were acquired for 100 s;
- delayed planar images (3 h p.i) were acquired for 141 s;
- late planar images (20 h p.i.) were acquired for 1007 s.

Images were acquired with a dual-head gamma camera (Forte, Philips) equipped with low-energy high-resolution collimators with energy window centred at 140 keV photopeak of [^99m^Tc] and a width of 20% by using a 512 × 512 matrix.

According to EANM guidelines [15], the time decay-corrected planar images were displayed in total number of counts using the same intensity colour scale both for delayed and late images, thereby avoiding operator bias in changing the intensity scale.

#### 2.1.3. Image Interpretation and Definition of Doubtful Cases

According to EANM guidelines [15], a scintigraphy was considered consistent for infection, when an increased activity, in terms of extent and/or intensity, was observed between delayed and late planar images in the region of interest (ROI). Conversely, the scintigraphy was considered negative if there was no uptake or a decreased activity over time in the ROI.

Doubtful cases at qualitative analysis of planar images, were defined and selected according to these criteria:

Small, not significant increase of activity, in the suspected region, at late images;

Increase of BM activity over time, that does not allow easy visualization of peri-prosthetic or bone activity:

- Small increase of extent of uptake in the suspected region, at late images, without significant increase of activity;
- Soft tissue infection that does not allow to clearly evaluate the bone;
- Small increase of activity over time, in the suspected region, but similar increase also in BM;
- Unmodified activity over time, in the suspected region, but with clear decrease of BM and background activity.

#### 2.1.4. Semi-Quantitative Analysis of Doubtful Cases

Equivocal cases were examined with a semi-quantitative analysis comparing the T/B ratios of delayed and late images and using contralateral site as background, as suggested in a large retrospective study [17].

Planar images were displayed on Hermes workstation and, by fixing a threshold of 40% of maximum pixel on late images, irregular ROIs were automatically drawn by the software on target and mirrored on contralateral background [17]. Same ROIs were applied to delayed images and mean counts per ROIs were recorded in order to calculate T/B ratios. In presence of BM interference on the evaluation of suspected area (criterion B), ROIs were manually drawn around the profiles of the whole prosthesis.

All doubtful cases were analysed by using 5 different thresholds (5%, 10%, 15%, 20% and 30%) of increase in T/B ratio over time, as criterion of positivity.

**Figure 1:**
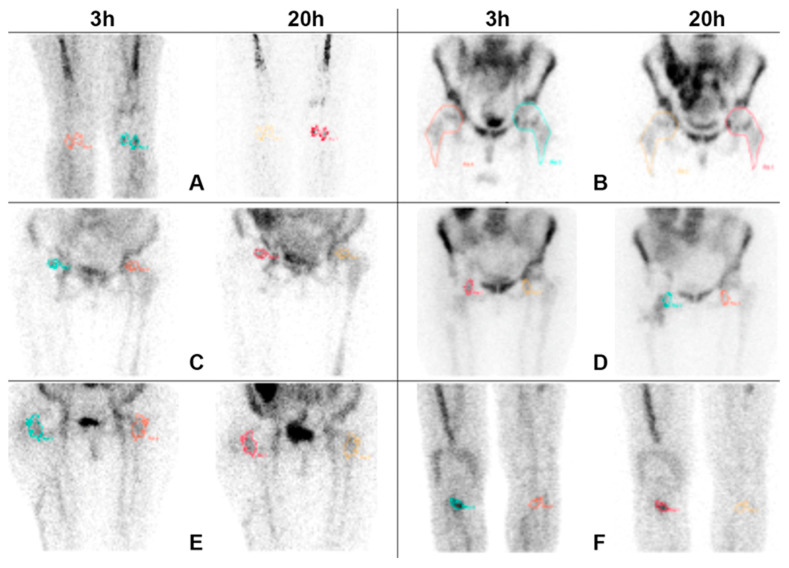
Anterior views acquired 3 h and 20 h after i.v. injection of radiolabelled leukocytes, with times corrected for isotope decay and displayed by using absolute counts and the same intensity colour scale. Images show an example of the methodology used for drawing regions of interests (ROIs) for all the criteria (**A**–**F**). In all doubtful cases, except for criterion B, irregular ROIs were automatically drawn on the target (T) of late images by fixing a threshold of 40% of maximum pixel, mirrored on the contralateral background (B) and copied on T and B of delayed images. For criterion B, due to the presence of bone marrow interference on the evaluation of the suspected area, ROIs were manually drawn on the whole prosthesis.

An example of ROIs analysis for each criterion is shown in Figure 1.

#### 2.1.5. Bone Marrow Scintigraphy (BMS)

BMS were performed in 56 patients by acquiring 300,000 counts in planar images of hips or knees approximately 30 min to 1 h after the i.v. administration of 370–500 MBq of [^99m^Tc]nanoalbumon (Radiopharmacy Laboratory Ltd., Budaörs, Hungary) and were compared with the images obtained with WBC scintigraphy. If the colloids distribution was spatially congruent with the activity observed at WBC scan (match), the exam was interpreted as physiological BM expansion, thus ruling out the infection. Conversely an activity detectable at WBC scintigraphy and not confirmed at BMS (mismatch), was consistent with an infection [19].

#### 2.1.6. Gold Standard

Final diagnosis was achieved with microbiology, obtained after surgery or during a diagnostic arthrocentesis, or histology, performed after bone biopsy.

In particular, the diagnosis of PJI was established when three or more intraoperative cultures (for both aerobic and anaerobic pathogens) or combination of preoperative arthrocentesis (with total cell count and differential leukocyte count and cultural examination) and intraoperative cultures provided the same micro-organism. Furthermore, the isolation of a pathogen in a single specimen of tissue biopsy was indicative of PJI (22).

An aseptic loosening was diagnosed following comprehensive clinical, microbiological/histological and imaging confirmation. In particular, patients with imaging suggestive for loosening and without the isolation of any pathogen were classified as having aseptic loosening.

Non-specific painful prosthesis was diagnosed in patients that did not show any infection or signs of dislocation of the device.

When biopsies or cultures were not available, a clinical follow-up of at least 18 months was used in order to confirm or rule out the diagnosis.

#### 2.1.7. Statistical Analysis

Statistical analysis was performed using SAS version 9.4 and JMP version 14 (SAS Institute, Cary, NC, USA). Continuous variables were presented in mean ± Standard Deviation (SD). The sensitivity, specificity and diagnostic accuracy of the techniques were calculated by SAS. Comparisons of the sensitivity, specificity and diagnostic accuracy between the techniques were performed using Z test for the equality of two proportions. Benjamini–Hochberg procedure was applied to check multiple comparisons. The results are reported in percentage with 95% confidence intervals (CIs). A *p*-value <0.05 was considered statistically significant.

## 3. Results

### 3.1. Patients

From January 2011 to December 2017, 871 [^99m^Tc]HMPAO-WBC scintigraphies (426 for hip prosthesis and 445 for knee prosthesis) were performed for suspected monolateral PJI in our Department. Twenty-eight patients were excluded because we could not retrieve final diagnosis. Therefore, 842 scintigraphies were analysed. In 159 cases (18.9%), qualitative analysis was consistent with an infection and in 614 (72.8%) it was clearly negative. The remaining 70 scintigraphies (8.3%) were considered equivocal by two or three readers. Amongst them, four patients were excluded for concomitant antibiotic therapy and possible false negativity.

**Figure 2:**
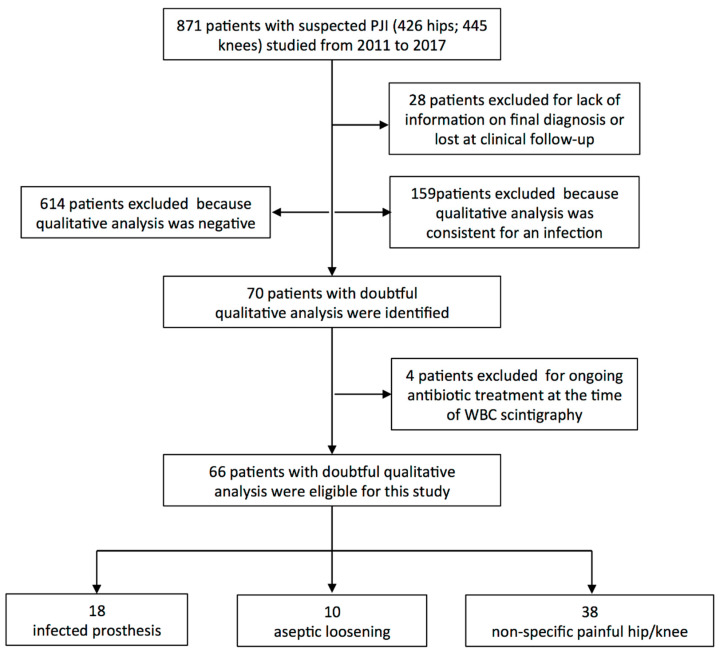
Flowchart of patients included in the study.

Therefore, 66 doubtful WBC scans were included in the present study and further evaluated with T/B analysis (Figure 2).

A descriptive analysis of our population is summarized in Table 1.

Patients were 20 males and 46 females. Time after surgery ranged between 1 up to 16 years (mean 4.28 ± 4.19) and mean age was 72.24 ± 10.42 years. Thirty-four patients had hip prosthesis and the remaining 32 had knee prosthesis.

Final diagnosis was achieved with surgery in 25 patients, with joint aspiration and microbiological examination in 28 patients and with clinical follow-up in the remaining 13 patients.

Based on diagnostic criteria previously described, an infection was detected in 18 patients (27.3%). Fifteen of them underwent to surgery and three were treated with antibiotics because not operable. An aseptic loosening was diagnosed in 10 patients (15.2%), by mean of biopsy in seven patients, and after surgery in the other three patients. Non-specific aseptic “painful prosthesis” was detected in the remaining 38 patients (57.5%) that were all treated with anti-inflammatory therapy or analgesics.

### 3.2. WBC Scintigraphy

Mean administered activity of [^99m^Tc]HMPAO ranged between 370 and 740 MBq (512.27 ± 156.14). Labelling efficiency (LE) ranged between 70% and 96%.

The distribution of patient population according to the different criteria at qualitative analysis and the number of additional BMS performed for each criterion is shown in Table 2.

### 3.3. Semi-Quantitative Analysis

The results of T/B ratios analysis, with 5 different thresholds, according to final diagnosis are shown in Table 3. By using the cut-off of 5% of increase over time, 41 out of 66 patients (62.12%) were classified as positive but only 14 of them were really infected, therefore showing high number of false positive (FP) cases. On the other hand, four patients with proven infection were not identified by this cut-off. This resulted in moderate sensitivity (77.8%) and low specificity (43.8%). By increasing the cut-off, the sensitivity drops down and the specificity grows in parallel, reaching the highest values with the cut-off at 30% of increase over time (93.8%). Intermediate thresholds showed similar sensitivity (72.2% for the cut-off at 10% and 66.7% for both 15% and 20%), but the highest specificity and accuracy were obtained when considering the increase of 20% between delayed and late images, as criterion of positivity (85.4% and 80.3%, respectively) (Table 3).

### 3.4. Bone Marrow Scintigraphy (BMS)

Fifty-six out of 66 patients with doubtful qualitative analysis also performed a BMS with [^99m^Tc]colloids.

Mean administered activity ranged between 185 and 370 MBq (229.92 ± 52.47).

**Figure 3:**
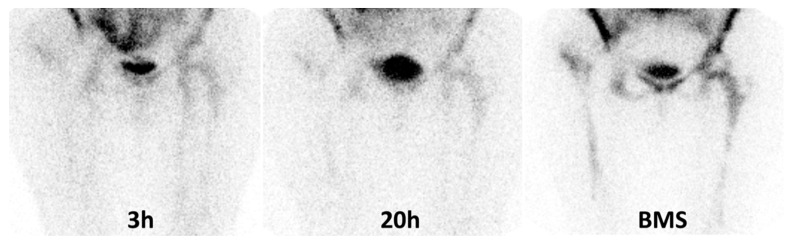
These images show an example of false negative BMS in a patient with histologically confirmed infection of right hip prosthesis. From the left to right: the uptake on the right trochanter at 3 h does not change after 20 h at visual analysis therefore her WBC scintigraphy was classified as doubtful according to criterion “A”. However, we observed an increase of more than 20% at semi-quantitative analysis. The patient performed a bone marrow scan (BMS) (image on the right panel) that showed a congruent uptake with WBC thus excluding the infection. After surgery, an infection by St. Aureus was detected.

Sensitivity, specificity and accuracy of BMS according to final diagnosis were 84.6%, 93.0% and 91.1%, respectively (Table 4). Figure 3 shows an example of false negative (FN) case at BMS.

### 3.5. Comparison between Semi-Quantitative Analysis and BMS according to Final Diagnosis

BMS showed significantly higher specificity and accuracy than cut-off at 5%–10% and 15% and significantly higher sensitivity than cut-off at 30%. However, combined approach did not provide statistically significant differences compared to the cut-off at 20% of increase over time (Table 5).

## 4. Discussion

Robust evidence in the literature exists on the role of radiolabelled WBC scintigraphy in detecting PJI [23]. The recently published EANM guidelines have definitely clarified all the aspects able to reach a high accuracy in detecting an infection by using this imaging modality [15]. Indeed, if correctly acquired, displayed and interpreted, planar images allow to accurately differentiation between an infection from a sterile inflammation, as it has also been demonstrated in other clinical scenarios [24]. However, despite these precautions, some scintigraphies still remain doubtful basing only on visual analysis.

In some cases, qualitative analysis of planar images may be equivocal for the presence of a slight increase of uptake and/or extent at late images. In these cases, different NM physicians could classify the same scintigraphy as positive or negative, depending on their subjective visual interpretation. Another possible source of doubt is the presence of BM expansion that is frequently observed after a prosthetic joint replacement and it is the result of a physiological migration of granulocytes in the reticulo-endothelial system. Over time, the activity of bone marrow may remain stable, increase or decrease similarly to the suspected area, thus interfering with the qualitative assessment. In these cases, an additional scintigraphy with colloids is strongly recommended in order to obtain a scintigraphic map of BM activity and to rule out or confirm the infection [19,20]. When the uptake of radio-colloids is spatially congruent with the activity observed at WBC scintigraphy, the hypothesis of an infection may be rejected, although FN cases may be observed in patients with small infection and concomitant physiological BM expansion (Figure 3). Conversely, in presence of a mismatch, the uptake shown at WBC should be considered consistent with an infection. The role of this combined approach is well established nowadays and allows reducing the number of FP cases due to BM activation thus maximizing the accuracy of WBC scintigraphy. The reported accuracy of this dual approach, in general population, ranges from 83% to 98% for both hip and knee prosthesis [19,20,25,26,27,28,29,30]; therefore, it is widely adopted in many centres despite the administration of an additional amount of radioactivity and an additional scan-time, which could result in being inconvenient for the patients.

Such a high diagnostic accuracy can also be achieved by using a correct image acquisition and interpretation procedure on planar gamma-camera images, and better if correlated by SPECT/CT [15,16,17] without the use of a combined BM scan.

However, the final diagnostic accuracy of WBC scan in a reported study can be highly influenced by the number of doubtful cases in the studied population. This is very rarely reported in scientific papers and may largely affects the final accuracy.

Aiming at achieving an accurate diagnosis and sparing additional radioactive doses to the patients, a semi-quantitative analysis of planar gamma-camera WBC images could be helpful in equivocal cases, but its role has not been systematically addressed and a precise cut-off of increase in T/B ratios over time still does not exist. Pelosi and colleagues in 2004, performed semi-quantitative evaluation in patients with suspected PJI using the left iliac crest as reference [18], thus requiring the inclusion of this region in the field of view in all scintigraphies. Of course, the choice of reference tissue, the size/shape and the placement of ROIs is operator-dependent and may strongly influence the results. In a large single-centre retrospective study on 297 patients with suspected muskoloskeletal infection (amongst which, 138 patients with PJI), authors analysed four different reference tissues: contralateral tissue, anterior superior iliac crest, ipsilateral bone marrow and contralateral bone marrow [17]. In this large series, the choice of contralateral tissue as reference provided the best results. However, they did not define precise thresholds for the increase in uptake over time. In 2014, Erba et al. retrospectively reviewed 235 WBC scintigraphies for suspected muskoloskeletal infection acquired with a fixed-time acquisition protocol and with a time corrected for isotope decay protocol [16]. They performed both qualitative and semiquantitative analysis by using different thresholds of increase in T/B ratios over time (5%; 10%, 20% and 25%) concluding that the best accuracy, sensitivity and NPV were obtained considering any percentage of increase of uptake over time.

In our study we applied automatic ROIs by fixing a threshold of 40% of maximum pixel on late images thus avoiding the operator bias and we found that, by progressively increasing the thresholds, the sensitivity decreases and specificity increases. As always, the best performance of a diagnostic test derives from a balance between sensitivity and specificity, but it also depends on the exact clinical indication. For example, for the assessment of antibiotic therapy efficacy after the diagnosis of an infection, we mainly need a sensitive test able to detect if the infection is still present or not. Therefore, for this particular purpose, we can speculate that the threshold of 5% could be used in order to select the highest number of TP patients that still require additional treatments. But it needs to be confirmed by larger studies.

On the other hand, in a diagnostic setting we need a good sensitivity, specificity and accuracy. In our population, the cut-off at 20% of increase of radioactivity over time showed the highest accuracy (80.3%) and a good compromise between sensitivity (66.7%) and specificity (85.4%). BMS showed significantly higher specificity and accuracy than cut-off at 5%, 10% and 15% and significantly higher sensitivity than cut-off at 30%, thus confirming the added value of this combined approach in doubtful cases but we did not find any significant difference comparing BMS and the cut-off at 20% of increase of radioactivity over time.

The best sensitivity and specificity achieved with a cut-off at 20% of increase of radioactivity over time (66.7% and 85.4%, respectively) are much lower than those generally reported for WBC scintigraphy, but we should underline that we analysed only doubtful cases at qualitative analysis.

This study has several limitations. First of all, not all the patients underwent surgery, bone biopsy or joint aspiration, since some clinicians preferred to perform a “wait and see” approach depending on the specific case. Therefore, in our study 13 patients were followed up for at least 18 months. With the aim to assess whether the inclusion of these patients could affect the sensitivity, specificity and accuracy of the different methods, we also performed a statistical analysis of data without including these 13 patients and we obtained the same results and reached same final conclusions.

Moreover, given the retrospective nature of this study, we could not be able to retrieve causative pathogen in all included patients and ten patients did not perform an additional BMS. However, our primary aim was to compare different thresholds of semi-quantitative analysis according to final diagnosis regardless to the aetiology. The secondary endpoint was to compare this method with BMS, that is commonly, but not necessarily in all cases, used in clinical practice.

Moreover, we did not analyse the added value of hybrid single photon emission computerized tomography/computerized tomography (SPECT/CT). Indeed, it is well known that the role of hybrid imaging is undeniable, and SPECT/CT can improve the localization and extent of infection [4,9,15,31,32,33,34]. However, the correct diagnosis of presence/absence of infection can be achieved by considering the increase of activity over time at planar images and indeed, in our Centre, SPECT/CT acquisitions are usually performed at 20 h (for 30–40 sec/step) only in patients with positive planar images, not for diagnostic purpose, but in order to achieve a better anatomic overview of the infective process and to provide an accurate information on its extent and localization into the bone or soft tissues. However, we cannot exclude that a SPECT/CT might be helpful in clarifying the presence or absence of infection in doubtful planar images in our population.

Finally, the choice of ROIs and their placement is operator-dependant; therefore, we used a software able to automatically draw irregular ROIs on the target and to mirror them on the contralateral background aiming to make the methodology more reproducible. However, this approach was not possible in patients with doubtful WBC according to criterion B for the interference of BM activity on the evaluation of prosthesis. In these eleven cases we manually drawn the ROIs around the profile of whole prosthesis that was clearly visible on planar images but, of course, we cannot exclude minimal differences among different operators, thus introducing a possible bias.

**Figure 4:**
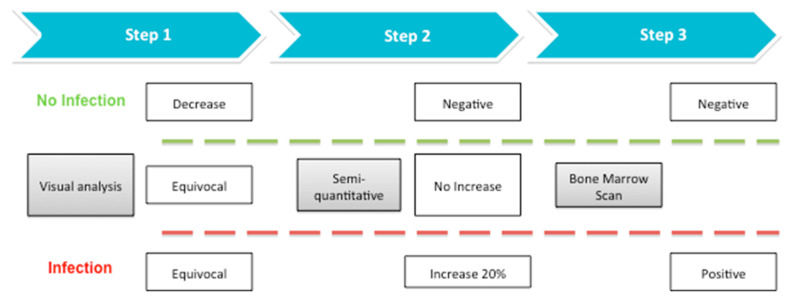
Proposed flow-chart for doubtful qualitative analysis of WBC scintigraphy.

Although no definitive conclusions may be drawn because of the small population, the cut-off at 20% seems to be a reliable tool to identify patients with an infection thus avoiding performing additional BMS. On the basis of these preliminary results, we propose a step-by step approach that is summarized in Figure 4. In case of doubtful qualitative assessment, a semi-quantitative analysis should always be performed before programming a BMS. If an increase of less than 20% in T/B ratios between delayed and late images is observed, the patient should perform a BMS in order to confirm or rule out the infection. Conversely, if we observe an increase of more than 20% between delayed and late images, we may diagnose an infection with high accuracy, thus avoiding an additional exam to the patient. Of course, larger retrospective and prospective studies are mandatory to confirm our results. We also foresee to explore the role of semi-quantitative analysis in SPECT/CT images and when using ^111^In-WBC instead of ^99m^Tc-WBC.

## 5. Conclusions

The management of doubtful cases at qualitative analysis of radiolabelled WBC scintigraphy still remains an important daily problem and often requires an additional BMS in order to confirm or rule out an infection. In equivocal cases, a semi-quantitative analysis should be attempted and the cut-off of more than 20% of increase of radioactivity over time might help in identifying infected patients from patients with aseptic prosthesis loosening.

## Figures and Tables

**Table 1:** Characteristics of study population.

	All Patients (66)	Infected (18)	Non Infected (48)
Age (years)	72.24 ± 10.42	71.56 ± 10.33	72.4 ± 10.4
Gender (F-M) *	69.7%–30.3%	61.1%–38.9%	72.9%–27.1%
Time after surgery (years)	4.28 ± 4.19	2.67 ± 1.37	4.8 ± 4.6
Type of prosthesis (Hip-Knee) *	51.5%–48.5%	50.0%–50.0%	50.0%–50.0%
T/B_3h_	1.67 ± 0.67	1.60 ± 0.56	1.69 ± 0.71
T/B_20h_	1.85 ± 0.74	2.00 ± 0.68	1.79 ± 0.75

All data are expressed in mean ± SD, except for gender and type of prosthesis that are reported in % (*); T/B: target/background ratio (using contralateral tissue as background).

**Table 2:** Distribution of patient population according to doubtful criteria.

Criteria for Doubtful Interpretation	*n*. of Patients	Patients with BMS
A. Small, not significant increase of activity in the suspected region at late images	36	30
B. Increase of bone marrow activity over time, that does not allow easy visualization of peri-prosthetic or bone activity	11	9
C. Small increase of extent of uptake in the suspected region at late images, without significant increase of activity	4	4
D. Soft tissue infection that does not allow to clearly evaluate the bone	6	5
E. Small increase of activity over time, in the suspected region, but similar increase also in bone marrow	2	2
F. Unmodified activity over time in the suspected region with clear decrease of bone marrow and background activity	7	6
	tot: 66	tot: 56

**Table 3:** Number of true positive, false positive, true negative and false negative patients detected by semi-quantitative analysis and BMS.

	*n*. TP	*n*. FP	*n*. TN	*n*. FN
T/B_20h_ > T/B_3h_ 5%	14	27	21	4
T/B_20h_ > T/B_3h_ 10%	13	16	32	5
T/B_20h_ > T/B_3h_ 15%	12	12	36	6
T/B_20h_ > T/B_3h_ 20%	12	7	41	6
T/B_20h_ > T/B_3h_ 30%	6	3	45	12
BMS	11	3	40	2

T/B: target/background ratio (using contralateral tissue as background); TP: true positive; FP: false positive; TN: true negative; FN: false negative; BMS: bone marrow scintigraphy.

**Table 4:** Overview of the performance of different diagnostic approaches in doubtful qualitative analysis.

	Sensitivity (%) (95% CI)	Specificity (%) (95% CI)	Accuracy (%) (95% CI)
T/B_20h_ > T/B_3h_ 5%	77.8 (58.6–97.0)	43.8 (29.7–57.8)	53.0 (41.0–65.1)
T/B_20h_ > T/B_3h_ 10%	72.2 (51.5–92.9)	66.7 (53.3–80.0)	68.2 (56.2–79.4)
T/B_20h_ > T/B_3h_ 15%	66.7 (44.9–88.4)	75.0 (62.8–87.3)	72.7 (62.0–83.5)
T/B_20h_ > T/B_3h_ 20%	66.7 (44.9–88.4)	85.4 (75.4–95.4)	80.3 (70.7–89.9)
T/B_20h_ > T/B_3h_ 30%	33.3 (11.6–55.1)	93.8 (86.9–100)	77.3 (67.2–87.4)
BMS	84.6 (65.0–100)	93.0 (85.4–100)	91.1 (83.6–98.5)

T/B: target/background ratio (using contralateral tissue as background); BMS: bone marrow scintigraphy; CI: confidence interval.

**Table 5:** Comparison between different thresholds of semi-quantitative analysis and BMS according to final diagnosis in terms of *p* values.

	Sensitivity (*p*)	Specificity (*p*)	Accuracy (*p*)
T/B_20h_ > T/B_3h_ 5% vs. BMS	0.63	**<0.0001**	**<0.0001**
T/B_20h_ > T/B_3h_ 10% vs. BMS	0.51	**0.005**	**0.005**
T/B_20h_ > T/B_3h_ 15% vs. BMS	0.43	**0.035**	**0.016**
T/B_20h_ > T/B_3h_ 20% vs. BMS	0.43	0.31	0.09
T/B_20h_ > T/B_3h_ 30% vs. BMS	**0.023**	0.89	0.06

T/B: target/background ratio (contralateral tissue); BMS: bone marrow scintigraphy; BMS: bone marrow scan; statistically significant differences are showed in bold.
